# Association of *FAAH* rs324420 (C385A) Polymorphism with High-Level Performance in Volleyball Players

**DOI:** 10.3390/genes14061164

**Published:** 2023-05-26

**Authors:** Hugo-Henrique Silva, Valéria Tavares, Maria-Raquel G. Silva, Beatriz Vieira Neto, Fátima Cerqueira, Rui Medeiros

**Affiliations:** 1 ICBAS-Institute of Biomedical Sciences, University of Porto, 4050-313 Porto, Portugal; valeria.tavares@ipoporto.min-saude.pt; 2 Portuguese Ministry of Education, 1399-025 Lisboa, Portugal; 3 Senior Rink-Hockey Team, Uniao Desportiva Oliveirense-Simoldes, 3720-256 Oliveira de Azemeis, Portugal; 4 Molecular Oncology and Viral Pathology Group, Research Center of IPO Porto (CI-IPOP)/RISE@CI-IPOP, Portuguese Oncology Institute of Porto (IPO Porto)/Porto Comprehensive Cancer Center (Porto.CCC), 4200-072 Porto, Portugal; i37300@ipoporto.min-saude.pt (B.V.N.); fatimaf@ufp.edu.pt (F.C.); ruimedei@ipoporto.min-saude.pt (R.M.); 5 FMUP-Faculty of Medicine, University of Porto, 4200-072 Porto, Portugal; 6 FP-I3ID, FP-BHS, University Fernando Pessoa, 4249-004 Porto, Portugal; 7 Faculty of Health Sciences, University Fernando Pessoa, 4200-150 Porto, Portugal; 8 CIAS-Research Centre for Anthropology and Health—Human Biology, Health and Society, University of Coimbra, 3000-456 Coimbra, Portugal; 9 CHRC-Comprehensive Health Research Centre, Nova Medical School, Nova University of Lisbon, 1150-090 Lisboa, Portugal; 10 Scientific Committee of the Gymnastics Federation of Portugal, 1600-159 Lisboa, Portugal; 11 CIIMAR/CIMAR, Interdisciplinary Centre of Marine and Environmental Research, 4450-208 Matosinhos, Portugal; 12 Pathology and Laboratory Medicine Department, Clinical Pathology SVIPO Porto Portuguese Oncology Institute of Porto, 4200-072 Porto, Portugal; 13 LPCC, Research Department, Portuguese League Against Cancer (LPPC—NRN), 4200-172 Porto, Portugal

**Keywords:** *FAAH*, high-level performance, volleyball, gene, polymorphism

## Abstract

Genetic variants are recognized to affect athletic performance, partially by modulating competition-facilitating behavior. In this study, the role of three genetic variants previously linked to athlete status was investigated among elite volleyball players. A total of 228 players (26.7 ± 8.1 years old) participating in the Portuguese championship and with multiple medalists in national and international competitions were evaluated in terms of anthropometrics, training regime, sports experience, and a history of sports lesions. SNP genotyping was conducted by means of TaqMan^®^ Allelic Discrimination Methodology. Volleyball players showed significantly different anthropometric indicators and training habits according to sex (*p* < 0.05). The A allele of the genetic variant *Fatty Acid Amide Hydrolase* (*FAAH*) rs324420 (C385A) was shown to be significantly associated with superior athletic achievements under a dominant genetic model (AA/AC vs. CC, odds ratio (OR) = 1.70; 95% Cl, 0.93–3.13; *p* = 0.026; *p* < 0.001 after Bootstrap), which was corroborated by a multivariable analysis (AA/AC vs. CC adjusted OR = 2.00; 95% Cl, 1.04–3.82; *p* = 0.037). Age and hand length were also found to be independently associated with high-level performance (*p* < 0.05). Our results confirm the role of *FAAH* in athletic performance. More investigation into this polymorphism’s potential impact on stress coping, pain, and inflammation regulation in sport, particularly in the scope of lesions prevention and treatment, is required.

## 1. Introduction

High-level performance athletes (i.e., elite athletes) present an extraordinary interindividual variability of physical but also mental traits that greatly influence their sports accomplishments and their career longevity [1,2,3]. In addition to training regimes [4] and optimal nutrition [5], individual genetic variation is thought to be another preponderant factor in elite athletic performance [1,2].

Over the years, several genes, including, for instance, *Angiotensin-I-Converting Enzyme* (*ACE)* and *Actinin α 3* (*ACTN3*), have been associated with athlete phenotypes and many physiological functions related to muscle-skeletal, respiratory, cardiovascular, nervous, and other systems implicated in sports practice [1,2,6]. However, genetic studies on competition behavior, stress management, and a potential relationship with sports lesions are scarce [2,3,6,7,8]. Recently, our research team has investigated the implications of the single-nucleotide polymorphism (SNP) *Fatty Acid Amide Hydrolase* (*FAAH*) rs324420 (C385A), among other genetic variants, in elite athletic performance [3]. Our results suggested that the SNP A allele (385A allele) was independently linked to enhanced performance of the world’s best rink-hockey players (dominant genetic model, AA/AC vs. CC; adjusted odds ratio (aOR) = 2.88; 95% Cl, 1.06–7.80; *p* = 0.038). The gene *FAAH* has been previously associated with pain tolerance, stress, and inflammation, which are features with implications for sports practice [9]. The role of the C385A variant in athletic performance is, however, not consensual, with evidence suggesting a detrimental effect of the 385A allele, as it seems to be more prevalent among inactive individuals than in elite athletes from different sports modalities [7,8]. As the cumulative evidence concerning the C385A variant is inconsistent, validation studies considering other sports modalities in addition to rink-hockey are a must to dissect the role of this SNP in athletic performance.

Volleyball is a high-intensity, intermittent sport modality with repetitive episodes of short durations of effort at diverse speed levels and incomplete recovery periods. Both aerobic and anaerobic metabolisms, as well as cardiovascular and musculoskeletal systems, are crucial for the performance of volleyball players [10]. The game is not time-limited. The team that wins the match is the team that first wins three sets or, in the case of a 2-2 tie, the team that wins the fifth set (deciding set) with at least 15 points and a minimum lead of 2 points (*Fédération Internationale de Volleyball*, FIVB—Volleyball International Federation). Regarding the substitution of players, an athlete may leave and re-enter the game, but only once in a set, occupying only his/her previous position in the line-up [11]. Volleyball is mostly described by its biomechanical component in the vertical plane due to the need for the ball to cross the vertical net to the opponent’s court. Although it is a non-invasion game, the frequency of sports injuries is high due to the inherently high training load and mechanical stress during the repetitive technical movements [10]. As such, mental abilities in the context of high-level performance are needed to manage stress and anxiety, be resilient, and avoid impulsivity to make optimal decisions during training sessions and competition [3,7,12]. This is particularly important in stressful moments of the athletes’ sports careers, namely, when they must demonstrate mental discipline and resilience (for instance, in the deciding fifth set of the volleyball match) or cope with sports lesions that may limit their mental and physical readiness to play [13]. In this context, the present study focuses on evaluating the impact of athlete status-related genetic markers with roles in brain function in a cohort of high-level performance volleyball players competing in the Portuguese national championship.

## 2. Materials and Methods

### 2.1. Enroled Players’ Description and Data Collection

A cohort of 228 volleyball players of high-level performance (i.e., involved in any national- or high-level team under the responsibility of a National Sports Federation) [3] competing in the Portuguese Volleyball Championship during three consecutives sports seasons (2019/2020, 2020/2021, and 2021/2022) were enrolled in this study. These players are currently the nation’s elite volleyball athletes, with many being multiple medalists in national and international competitions.

For participants’ selection, the inclusion criteria were the following: (1) be an athlete at least 18 years old at the national minimum level, (2) be an experienced national athlete (i.e., considered a world’s elite athlete competing in Portugal), and (3) be a competitor that has reached a higher level (i.e., World, European, and Continental Championships or international competitions such as the Olympic Games) [3].

At the time of recruitment, the athletes’ sport experience was 16.3 ± 8.4 years; their training frequency was 6.5 ± 1.5 days per week and 3.3 ± 1.0 h per day, for a total of 22.0 ± 10.5 h per week. The characterization of the enrolled athletes is shown in Table 1.

Each participant in the study signed an informed consent prior to their recruitment and this study was approved by the Ethical Committee of the University Fernando Pessoa at Porto (CEUFP05062017).

Data on anthropometrics, training conditions, and sports experience, as well as the history of sports lesions, were assessed by the same trained researcher via questionnaires that were personally distributed to the players prior to training sessions. Determination of height, body mass index (BMI), waist circumference (WC), hip circumference (HC), waist/hip ratio (WHR), hand length, and shoe size were described elsewhere [14]. In terms of training regime, training hours per day and training units per week were assessed to determine the number of training hours per week. Athletes’ national and international achievements, as well as their participation in national and regional teams were also assessed. The history of sports lesions was investigated, including the anatomical part affected, the year of occurrence, and the time of recovery from the first severe injury [3,5].

#### 2.1.1. Biological Sample Processing

Buccal cell samples from each athlete were collected from both right and left using sterile swabs (FL Medical, Hamburg, Germany) [15]. Genomic deoxyribonucleic acid (DNA) was extracted from these samples using QIAamp DNA Blood Mini Kit, Qiagen. Purity (A260/A280) and concentration of the DNA samples were evaluated using NanoDrop Lite spectrophotometer (Thermo Scientific^®^, Waltham, MA, USA). Only samples with DNA concentration of at least 4 ng/µL and with A260/A280 between 1.70 and 2.00 were used for SNP genotyping.

#### 2.1.2. SNP Selection and Genotyping

In a previous literature review conducted by our research group on genetic factors linked to athletic performance traits (power, endurance, and/or sports lesions) across different sports modalities [2], genetic variants were selected if (1) they were previously reported to influence athletic performance in a Caucasian population; (2) they had roles in brain activity; and (3) their minor allele frequency (MAF) in Iberian population was higher than 10% with the purpose of ensuring genotype representation. Furthermore, the selection was also based on the functional consequence of the genetic variants and the availability of TaqMan^®^ assays (Applied Biosystems). Applying these criteria, three genetic polymorphisms were selected (Table 2). SNP genotyping was conducted in a Real-Time Polymerase Chain Reaction (RT-PCR) system (Applied Biosystems) via TaqMan^®^ Allelic Discrimination methodology. All RT-PCR reactions were performed in 6 μL volumes with 2.5 μL of TaqMan^TM^ Genotyping Master Mix (2×), 2.375 μL of nuclease-free water, 0.125 μL of 40× TaqMan^®^ SNP Genotyping Assay (Table 2), and 1 μL of genomic DNA. Amplification conditions included the following: 95 °C for 10 min, 45 cycles of 95 °C for 15 s, and 60 °C for 1 min [16]. Two negative controls were included in all PCR runs, and replicates were performed on at least 20% of randomly selected samples (accuracy superior to 99%). Results were assessed independently by two researchers who had no prior knowledge of the athletes’ characteristics.

### 2.2. Statistical Analysis

Statistical analyses were performed using SPSS software version 26.0 (SPSS, Inc., Chicago, IL, USA). Continuous variables were described as mean ± standard deviation (SD), while categorical data were presented as frequencies. The categories of the variables age (≥26 versus (vs.) <26 years (years)), hand length (≥23 vs. <23 cm), WHR (≥0.85 vs. <0.85) and recovery time from the first severe sports lesion (≥10 vs. <10 months) were defined based on the median value. Associations between the genetic variants and the athlete features were analyzed by the student’s *t*-test for continuous variables (assuming normal distribution based on the cohort size), whereas the chi-square test (χ2) or Fisher’s exact test was used for categorical ones. The athletes’ accomplishments or success were determined by the total number of national and international player titles. Tertiles were used to classify athletic performance. In the highest tertile (those with more than 11 titles), athletes were regarded as super athletes (*n* = 62), while the two remaining tertiles were combined in one category regarded as others (*n* = 166) for further analyses. Univariate analyses were performed using a binomial regression model to identify SNPs that were predictive of superior athletic achievements. Recessive and dominant genetic models were considered for each genetic polymorphism in the univariate analyses. Only those with statistical significance were presented. Statistical power calculations concerning the univariate binomial regression analyses were performed using the software GPower version 3.1.9.7 (Heinrich Heine University, Düsseldorf, Germany) [20]. Relevant genetic polymorphisms and other relevant variables were further included in a multivariate analysis. Bootstrapping analyses were also conducted using Monte Carlo simulation (1000 replications). All tests, including the statistical power analysis, were two-tailed, and statistical significance was defined as *p* < 0.05.

## 3. Results

### 3.1. High-Level Performance Volleyball Players

Female volleyball players were younger (*p* = 0.026) and, as expected, demonstrated significantly lower anthropometric indicators (body mass, height, BMI, waist, hip circumferences, WHR, hand length, and shoe size) than male athletes (*p* < 0.01, Table 1). Female players also trained for significantly more days and hours per week and began earlier (16.2 ± 1.4 years old) at the high-level performance level than males (17.2 ± 2.0 years old, *p* < 0.01, Table 1), who were more experienced (17.0 ± 9.0 years vs. 14.8 ± 6.6 years) and had a higher number of participations in regional and national teams (28.2 ± 39.8 participations vs. 25.5 ± 37.9 participations), although significant differences were not found (*p* < 0.05). In addition, more than half of the volleyball players reported a high prevalence of sports lesions (63.8% males and 60.6% females), and the first severe sports lesion occurred at early ages (females, 11.8 ± 9.5 years old and males, 12.2 ± 9.0 years old), mostly in the second trimester of the year (5.5 ± 4.2 months in males and 6.5 ± 4.1 months in females, Table 1). Additionally, more than a quarter of the participants were professional volleyball players.

### 3.2. SNP Genotype Frequencies 

The genotypes’ distribution of each genetic variant by sex among the players is presented in Table 3.

### 3.3. Univariable and Multivariable Analyses

In the univariable analyses (*n* = 219, 59 super athletes and 160 others), *FAAH* rs324420 under a dominant genetic model (AA/AC (*n* = 85) vs. CC (*n* = 134)) was the only genetic variant significantly associated with sports achievements among high-level performance volleyball players (AA/AC vs. CC; OR = 1.70; 95% Cl, 1.13–3.13; *p* = 0.026) (*p* < 0.001 after Bootstrapping analyses were conducted using a Monte Carlo simulation for 1000 replications). It must be noted, however, that the study was only sufficiently powered (power ≥ 0.80) to detect odds ratio (OR) values of at least 1.70, considering the entire cohort with available information for SNP genotypes (*n* = 219).

The effect of *FAAH* rs324420 was confirmed by a multivariate analysis (*n* = 219) adjusted for age, sex, BMI, hand length, and history of severe sports lesions (AA/AC vs. CC; aOR = 2.00; 95% Cl, 1.04–3.82; *p* = 0.037; Table 4). In the multivariable analysis, *FAAH* rs324420, age, and hand length were independent predictors of high athletic achievements among elite volleyball players. In accordance with previous findings, the 385A allele appears to be beneficial since the AA or AC genotype carriers were two times more prone to be super athletes than players carrying the CC genotype. Additionally, as expected, in the study, older players (≥ 26 vs. < 26 years; aOR = 2.96; 95% Cl, 1.55–5.66; *p* = 0.001; Table 4) and the ones with bigger hands (≥ 23 vs. < 23 cm; aOR = 5.51; 95% Cl, 1.14–26.59; *p* = 0.034; Table 4) were found to be more prone to greater achievements.

## 4. Discussion

The influence of genes implicated in the activity of the brain regions associated with psychological traits is poorly explored among elite athletes [3,7]. In addition, the diversity of sports disciplines has made it difficult to detect the potential effect of genetic variants on athletic performance.

To the best of our knowledge, this is the first study investigating the role of genetic factors in the high-level performance of volleyball athletes in a homogeneous and unique cohort of elite subjects. Previous studies were mostly performed on diverse sporting disciplines with a reduced number of volleyball athletes, mostly males, and the potential interaction between the athlete’s phenotype and biological systems was not consistently observed [3]. Briefly, a cross-sectional study with 131 elite Turkish athletes, including 33 volleyball players (20.8 ± 4.6 years old, 1.97 ± 2.3 m, 91 kg and 23.3 ± 3.1 kg/m^2^) found a co-beneficial effect of the *ACTN3* R577X (rs1815739) X allele (RX vs. RR, OR = 5.01, 95% CI, 2.45–10.2; *p* < 0.0011; XX vs. RR, OR = 5.06; 95% CI, 2.04–12.59; *p* < 0.0013; RR vs. RX + XX, OR = 0.19; 95% CI, 0.1–0.38; *p* < 0.0011). Although the volleyball players were considered elite athletes by Dogan and co-workers [21], their training regime and sex were not clearly disclosed. In addition, a study concerning the association between *ACTN3* rs1815739 and the *ACE* I/D gene variation with changes in blood pressure of 107 Serbian Caucasian male players (athletes in mixed sports: volleyball, water polo, and handball (*n* = 54); endurance athletes from middle-distance swimmers, football players. and rowers (*n* = 36); sprint/power from swimmers < 200 m, and short-distance runners (*n* = 17) found that the lowest maximal systolic blood pressure was presented by carriers of *ACTN3* rs1815739 CC genotype. In opposition, the highest percentage of systolic blood pressure decline after the maximal incremental stress test was shown by those carrying the *ACE* DD genotype [22]. Although the *ACE* insertion (I allele) has been related to increased endurance performance in elite athletes [23], this Serbian study did not confirm this effect in various sports, including volleyball [20]. More recently, a study conducted by Orysiak and co-workers [24] with Polish Caucasian female (15.8 ± 2.0 years old and 6.6 ± 2.1 years of sport experience; *n* = 132) and male (16.7 ± 2.1 years old and 7.7 ± 2.9 years of sport experience; *n* = 266) athletes from different sports modalities (volleyball, canoeing, swimming, and ice hockey) found that the distribution of genotypes of *ACTN3* and *ACE* genetic factors was not significantly different between athletes’ groups of both sexes. Additionally, the polymorphisms’ genotypes (in combination or alone) were not associated with the sum of mechanical power, muscle strength, and height of jump for athletes of both sexes [24,25]. Similarly, Ruiz and co-workers [26] did not observe any association between the *ACTN3* rs1815739 and the likelihood of being an elite volleyball player using either a recessive model (RR and RX vs. XX) or a dominant model (RR vs. RX and XX).

The scarce genetic research involving volleyball players has not solidified the role of neurotransmission and nerve plasticity in athletic performance. Although not confirmed, the dopamine neurotransmitter has been suggested to play a key role in high-level performance due to the athlete’s ability to develop emotional and psychological control [27]. However, this has not been confirmed. In a study with fifty elite athletes (involving six volleyball players) and one hundred unrelated healthy controls, genes involved in muscle development (*Myostatin* (*MSTN*)) and the management of aggressiveness, anxiety, and fear (*Solute Carrier Family 6 Member 4* (*SLC6A4*), *Solute Carrier Family 6 Member 3* (*SLC6A3*)*,* and *Monoamine Oxidase A* gene (*MAOA*)) were evaluated. No association of elite performance with muscle development regulation or the serotonin pathway was found [28].

The literature concerning the gene–environment interaction and sports lesions among volleyball players is also limited [29,30,31,32]. In a study involving Brazilian volleyball players (146 and 125 players, with and without tendinopathy, respectively; *n* = 271), the *Forkhead Box P3 (FOXP3*) rs3761549 and the *Fc Receptor Like 3 (FCRL3*) rs7528684 were studied for tendinopathy risk [32], which was more prevalent in males (OR = 2.87; 95% CI = 1.67–4.93) and related to increased age (OR = 8.75; 95% CI, 4.33–17.69) and volleyball experience (OR = 8.38; 95% CI, 3.56–19.73). Salles and co-workers [32] found that athletes carrying the *FCRL3* rs7528684 variant had a higher risk of developing tendinopathy (OR = 1.44; 95% CI = 1.02–2.04). A genotype/phenotype study examined the effect of the VDR-FokI polymorphism in 60 Italian athletes (35 males and 25 females; 11.7% volleyball players) with and without low back pain. Athletes carrying the F allele were two times more likely to develop low back pain (aOR = 2.55, 95% CI 1.02–6.43, *p* = 0.046) [13].

Given the current gaps in our knowledge, the present study was designed to assess the impact of three brain function-related genetic polymorphisms on the sports achievements of elite volleyball players. As expected, significant differences in the anthropometric profile between female and male athletes were shown, which could be due to biological-related factors. More than a quarter of the players were competing at a professional level, and a high incidence of sport-related lesions occurring at very early ages was reported by athletes of both sexes. Age, hand length, and the SNP *FAAH* rs324420 were independent predictors of high sports achievements. Regarding the impact of age, as older players have more sports experience, inevitably they are more prepared for the demands of high-level athletic performance. However, this might also be a mere case of opportunity, meaning older players had more opportunities to win competencies than their counterparts. As for the impact of hand length, this might be due to body composition requirements for technical skills in volleyball (e.g., serving, setting, spiking, and blocking) [32]. Thus, players with bigger hands are more equipped for the demands of volleyball.

**Figure 1:**
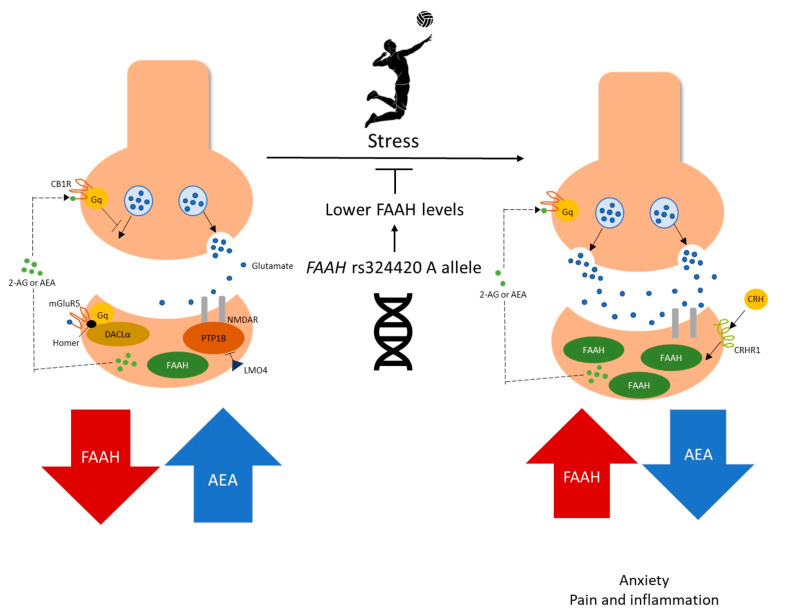
The central role of the endocannabinoid system in regulating the stress response of high-level performance volleyball players. In normal conditions, the endocannabinoid system represses the release of the neurotransmitter glutamate via N-arachidonoylethanolamine (AEA), modulating the synaptic function. Nevertheless, under acute stress, the mechanism mediated by corticotropin-releasing hormone (CRH) and its receptor (corticotropin-releasing hormone receptor 1-CRHR1) can be triggered and increase the fatty acid amide hydrolase (FAAH) activity in the basolateral amygdala. Consequently, AEA levels decrease, which are no longer able to restrain glutamate release. As a result, increased neural excitability in the basolateral amygdala enhances anxiety-like behavior (adapted by Silva et al.) [3]. CB1R: cannabinoid type 1 receptor; Gq: family G protein; mGluR5: metabotropic glutamate receptor 5; 2-AG: 2-arachidonoyl glycerol; DAGLα: diacylglycerol lipase-α; PTP1B: protein tyrosine phosphatase 1B; NMDAR: NMDA receptor; LMO4: LIM domain only 4.

The fatty acid amide hydrolase protein, involved in the metabolism of endocannabinoids, is encoded by the *FAAH* gene, which contains the SNP rs324420. The protein is recognized for breaking down the metabolite N-Arachidonoyl ethanolamide (Anandamide, AEA), which activates the cannabinoid type 1 receptor (CB1). The protein FAAH is activated during stress exposure circumstances (Figure 1), which raises the neuronal excitability in the amygdala, a critical brain area that mediates anxiety [7]. As opposed to this, FAAH inhibition lessens anxiety-like behavior [33] and may promote an antidepressant impact via stimulation of the CB1 receptor [34].

According to the literature, FAAH is a good candidate for drug discovery in patients dealing with pain and inflammation [35]. For elite athletes, especially those who play volleyball at a high level of performance with a high incidence of sports lesions, this is of vital importance. Prior research suggested that the 385A allele was associated with reduced FAAH levels [33], with prevalence rates of 16% and 37% in the populations of Africa and the Iberian Peninsula [36]. Although the resulting protein exhibits typical catalytic properties, the 385A allele is associated with a higher sensitivity of FAAH to proteolytic degradation and a shorter half-life, which accounts for the protein’s lower amounts [3,7]. The 385A allele may be linked to accelerated amygdala reactivity to threat habituation, enhanced learning of how to overcome fear, and diminished anxiety-like behavior. This is crucial for athletes to respond and adapt to unpredictably stressful situations, handle stress more effectively, and increase motivation for performing and competing at a high level [8,33,37]. This supports our results that showed that athletes carrying the 385A allele (AA or AC genotype) were twice as likely to be super athletes than those carrying the CC genotype (aOR = 2.00; 95% Cl, 1.04–3.82; *p* = 0.037) [3]. Furthermore, the A allele was also previously found to be associated with better athletic achievements among elite rink-hockey players (aOR = 2.88; 95% Cl, 1.06–7.80). Therefore, the well-noticed effect of *FAAH* rs324420 in high-level performance sports brings new insights for future research in the FAAH’s mechanism of action via the endocannabinoid system [38], applied for inflammation control, and pain regulation. This may also highlight the need to improve not only the athletes’ physical health and performance, but also their mental status, especially under stressful conditions [33].

As for the other two SNPs, in opposition to previous studies on athletic performance [13,27,39], no significant association was identified. However, it should be noted that the present study was not sufficiently powered to detect small effects. Hence, future studies with larger cohort sizes are required to better explore their impact. Briefly, the *Adrenoceptor Beta 2* (*ADRB2*) rs1042713 (Arg16Gly) A allele was previously associated with a lower resting cardiac output, protein density, and endurance performance [40], while the G allele was related to the sprint performance of young football players [41]. The *ADRB2* encodes the β2-adrenergic receptor (a member of the G protein-coupled receptor superfamily), which is primarily responsible for the regulation of many physiological systems with direct implications for athletic performance, including the central nervous, vascular, cardiac, pulmonary, and endocrine systems [41,42,43]. The protein is involved in the catecholamine system, namely, in the regulation of lipid mobilization from the adipose tissue, glucose uptake, and energy expenditure [40]. As for *Nitric Oxide Synthase 3* (*NOS3*) rs1799983 (G894T), it has been associated with the athletes’ susceptibility to sports lesions [44]. The SNP has also been shown to have a potential role in reducing blood pressure in response to physical exercise through the mechanism of endothelium vasodilatation, with implications for athletes’ endurance performance [27]. Additionally, athletes carrying the SNP T allele have shown a higher right ventricular mass index (32 ± 6 g vs. 27 ± 6 g, *p* < 0.01) and a larger right ventricular stroke volume index (71 ± 10 mL vs. 64 ± 10 mL, *p* < 0.01) than their counterparts (GG genotype carriers) [45].

Regarding the limitations of our study, although our cohort is highly representative of elite volleyball players competing in Portugal, the small cohort size may have limited the statistical power to detect small effects. Indeed, the sample collection for the present study was extremely affected by the COVID-19 pandemic-imposed restrictions, which also made it difficult to collect data on factors known to affect sports achievements [4]. Beyond additional studies with larger cohort sizes, further investigation on the effect of *FAAH* rs324420 on athletic performance should also include control groups (sedentary individuals and not elite athletes) to better assess the implications of this SNP in sports practice.

## 5. Conclusions

Elite volleyball players carrying the 385A allele seem to be more prone to be super athletes, which may be attributed to better stress-coping abilities and a higher pain tolerance. This result is in line with our previous findings concerning rink-hockey players of high-level performance. Considering the cumulated data, the SNP *FAAH* rs324420 may help coaches and clinical staff plan the athletes’ training, considering their individual mental and physical characteristics that together influence pain tolerance and decision-making in a competition. Thus, sports healthcare professionals, including medical doctors, nutritionists, and physiotherapists, should be aware of the potential of *FAAH* rs324420. However, despite the encouraging results concerning this SNP, additional studies with larger cohort sizes are mandatory to replicate and validate these findings and better characterize the functionality of *FAAH* rs324420. Inclusively, further investigation in other individual sports disciplines is encouraged. Additionally, this genetic polymorphism should also be investigated in the framework of sports lesion prevention, treatment/rehabilitation, and the return of volleyball players to sports activity. As for the other tested SNPs, their implications should be evaluated using larger sample sizes given the lack of study power to discover small effects.

## Figures and Tables

**Table 1:** Demographic, sports, and training characteristics of high-level performance volleyball players by sex (*n* = 228; 66 females and 162 males).

Variables	Total (*n* = 228)	Females (*n* = 66)	Males (*n* = 162)	*p*-Value
Age (years) *	26.7 ± 8.1	24.9 ± 6.1	27.5 ± 8.7	**0.026**
Body mass (kg) *	81.4 ± 12.2	69.4 ± 7.5	86.3 ± 10.2	**<0.001**
Height (m) *	1.86 ± 0.10	1.77 ±0.07	1.90 ± 0.09	**<0.001**
BMI (kg/m^2^) *	23.4 ± 2.1	22.2 ± 1.6	23.9 ± 2.0	**<0.001**
Waist circumference (cm) *	80.3 ± 6.0	75.4 ± 5.2	82.2 ± 5.1	**<0.001**
Hip circumference (cm) *	91.5 ± 10.0	83.4 ± 9.6	94.8 ± 7.5	**<0.001**
WHR *	0.88 ± 0.05	0.85 ± 0.04	0.91 ± 0.05	**<0.001**
Hand length (cm) *	25.0 ± 1.4	24.2 ± 1.5	25.3 ± 1.2	**<0.001**
Shoe size *	44.0 ± 3.1	40.2 ± 1.7	45.6 ± 2.1	**<0.001**
Age at first sports lesion (years) *	12.1 ± 9.1	11.8 ± 9.5	12.2 ± 9.0	0.803
Month of the first sports lesion *	5.8 ± 4.2	6.5 ± 4.1	5.5 ± 4.2	0.202
Beginning at high-level performance (years) *	16.9 ± 2.0	16.2 ± 1.4	17.2 ± 2.0	**<0.001**
Training experience (years) *	16.3 ± 8.4	14.8 ±6.6	17.0 ± 9.0	0.936
Training frequency *				
Days/week	6.5 ± 1.5	6.8 ± 1.6	6.4 ± 1.5	**<0.001**
Hours/day	3.3 ± 1.0	3.2 ± 1.0	3.3 ± 1.0	**0.004**
Hours/week	22.0 ± 10.5	23.0 ± 12.9	21.6 ± 9.3	**<0.001**
Participations in regional and national teams *	27.4 ± 39.2	25.5 ± 37.9	28.2 ± 39.8	0.778
Sport lesion				
No	85 (37.1)	26 (39.4)	59 (36.2)	0.381
Yes	144 (62.9)	40 (60.6)	104 (63.8)	
Nationality				
Portuguese	172 (75.1)	50 (75.8)	122 (74.8)	0.356
Others	57 (24.9)	16 (24.2)	41 (25.2)	
Ethnicity				
Caucasian	208 (90.8)	63 (95.5)	145 (89.0)	0.094
Others	21 (9.2)	3 (4.5)	18 (11.0)	
Other occupation/profession				
Athletes	69 (30.1)	20 (30.3)	49 (30.1)	0.471
Student	99 (43.2)	30 (45.5)	69 (42.3)	
Teacher	22 (9.6)	4 (6.1)	18 (11.0)	
Physical therapist	8 (3.5)	3 (4.5)	5 (3.1)	
Sport coordinator	1 (0.4)	--	1 (0.6)	
Coach	3 (1.3)	--	3 (1.8)	
Podiatrist	3 (1.3)	2 (3.0)	1 (0.6)	
Architect	1 (0.4)	1 (1.5)	--	
Engineer	7 (3.1)	3 (4.5)	4 (2.5)	
Market trader	8 (3.5)	1 (1.5)	7 (4.3)	
Other	8 (3.5)	2 (3.0)	6 (3.7)	

Bold values were regarded as significant (*p* < 0.05). * Variables described as mean ± standard deviation. BMI: body mass index; WHR: waist circumference/hip circumference ratio.

**Table 2:** Description of the evaluated genetic polymorphisms and the TaqMan^®^ assays used.

SNP	Functional Consequence	Biological Functions	TaqMan^®^ SNP Genotyping Assays ID
*ADRB2* rs1042713	Missense	Catecholaminergic system	C___2084764_20
*FAAH* rs324420	Missense	Neural functions, including nerve plasticity	C___1897306_10
*NOS3* rs1799983	Missense	Neurotransmission, antimicrobial, and antitumoral activities	C___3219460_20

SNP functional consequence was characterized based on the Ensembl database [17], and the encoded proteins’ biological functions were provided according to the GeneCards database [18] and UniProt database [19].

**Table 3:** Genotype distribution of the evaluated genetic variants among high-level performance volleyball players (*n* = 219; 60 females and 159 males).

Genotype Frequencies	Females (*n* = 66)	Males (*n* = 162)	Total (*n* = 219) *	*p*-Value
***ADRB2* rs1042713**				
AA	13 (21.7)	30 (18.9)	43 (19.6)	0.127
AG	19 (31.7)	74 (46.5)	93 (42.5)	
GG	28 (46.7)	55 (34.6)	83 (37.9)	
***FAAH* rs324420**				
AA	3 (5.0)	8 (5.0)	11 (5.0)	0.996
AC	20 (33.3)	54 (34.0)	74 (33.8)	
CC	37 (61.7)	97 (61.0)	134 (61.2)	
***NOS3* rs1799983**				
TT	10 (16.7)	24 (15.1)	34 (15.5)	0.196
GT	30 (50.0)	61 (38.4)	91 (41.6)	
GG	20 (33.3)	74 (46.5)	94 (42.9)	

* Polymorphism genotyping was not possible in 9 participants (6 females and 3 male athletes, respectively) resulting in a failed genotyping of 4%.

**Table 4:** Multivariate analysis on the elite volleyball players’ achievements (*n* = 219, 59 super athletes and 160 others) using binomial regression.

*Factors*	aOR	95% CI	*p*-Value
***FAAH rs324420***	2.00	**1.04–3.82**	**0.037**
*(AA/AC* vs. *CC*^1^*)*			
**Age**	2.96	**1.55–5.66**	**0.001**
(≥ 26 vs. < 26 years ^1^)			
**Sex**	0.82	0.37–1.82	0.620
*(Male* vs. *female*^1^*)*			
**BMI**	1.16	0.51–2.64	0.725
(≥ 25 vs. < 25 kg/m^2 1^)			
**Hand length**	5.51	**1.14–26.59**	**0.034**
(≥ 23 vs. < 23 cm ^1^)			
**History of sport lesions**	1.34	0.68–2.63	0.400
*(Yes* vs. *no* ^1^*)*			

Bold values were regarded as significant (*p* < 0.05). ^1^ Reference group. BMI, body mass index; CI, confidence interval; aOR, adjusted odds ratio.

## Data Availability

The authors confirm that the data supporting the findings of this study are available within the article.
